# Characterizing and targeting *PDGFRA* alterations in pediatric high-grade glioma

**DOI:** 10.18632/oncotarget.11602

**Published:** 2016-08-25

**Authors:** Carl Koschmann, Daniel Zamler, Alan MacKay, Dan Robinson, Yi-Mi Wu, Robert Doherty, Bernard Marini, Dustin Tran, Hugh Garton, Karin Muraszko, Patricia Robertson, Marcia Leonard, Lili Zhao, Dale Bixby, Luke Peterson, Sandra Camelo-Piragua, Chris Jones, Rajen Mody, Pedro R. Lowenstein, Maria G. Castro

**Affiliations:** ^1^ Department of Pediatrics, Division of Pediatric Hematology-Oncology, University of Michigan School of Medicine, Ann Arbor, MI 48109, USA; ^2^ Department of Neurosurgery, University of Michigan School of Medicine, Ann Arbor, MI 48109, USA; ^3^ Divisions of Molecular Pathology and Cancer Therapeutics, Institute of Cancer Research, London, SM2 5NG, UK; ^4^ Department of Pathology, University of Michigan School of Medicine, Ann Arbor, MI 48109, USA; ^5^ Department of Pharmacology, University of Michigan School of Medicine, Ann Arbor, MI 48109, USA; ^6^ Department of Pediatrics, Division of Neurology, University of Michigan School of Medicine, Ann Arbor, MI 48109, USA; ^7^ Department of Biostatistics, University of Michigan School of Medicine, Ann Arbor, MI 48109, USA; ^8^ Department of Internal Medicine, University of Michigan School of Medicine, Ann Arbor, MI 48109, USA; ^9^ Department of Cell and Developmental Biology, University of Michigan School of Medicine, Ann Arbor, MI 48109, USA

**Keywords:** pediatric high-grade glioma, PDGFRA amplification, PDGFRA mutation, brain tumor, tyrosine kinase inhibitor

## Abstract

Pediatric high-grade glioma (HGG, WHO Grade III and IV) is a devastating brain tumor with a median survival of less than two years. *PDGFRA* is frequently mutated/amplified in pediatric HGG, but the significance of this finding has not been fully characterized. We hypothesize that alterations of *PDGFRA* will promote distinct prognostic and treatment implications in pediatric HGG. In order to characterize the impact of *PDGFR* pathway alterations, we integrated genomic data from pediatric HGG patients (n=290) from multiple pediatric datasets and sequencing platforms. Integration of multiple human datasets showed that *PDGFRA* mutation, but not amplification, was associated with older age in pediatric HGG (P= <0.0001). In multivariate analysis, *PDGFRA* mutation was correlated with worse prognosis (P = 0.026), while *PDGFRA* amplification was not (P = 0.11). By Kaplan-Meier analysis, non-brainstem HGG with *PDGFRA* amplification carried a worse prognosis than non-brainstem HGG without *PDGFRA* amplification (P = 0.021). There were no pediatric patients with *PDGFRA*-amplified HGG that survived longer than two years. Additionally, we performed paired molecular profiling (germline / tumor / primary cell culture) and targeting of an infant thalamic HGG with amplification and outlier increased expression of *PDGFRA*. Dasatinib inhibited proliferation most effectively. In summary, integration of the largest genomic dataset of pediatric HGG to date, allowed us to highlight that *PDGFRA* mutation is found in older pediatric patients and that *PDGFRA* amplification is prognostic in non-brainstem HGG. Future precision-medicine based clinical trials for pediatric patients with *PDGFRA*-altered HGG should consider the optimized delivery of dasatinib.

## INTRODUCTION

Pediatric high-grade glioma (WHO Grade III and IV) is a devastating brain tumor carrying a poor prognosis. Despite histologic similarities to adult high-grade glioma (HGG), there are important differences from tumors arising in younger patients. In particular, pediatric HGG arises in different locations, more often in midline structures such as the brainstem and thalamus. As well, pediatric high grade gliomas almost always develop *de novo* as high-grade lesions, as opposed to secondary GBM seen in some adult patients [1–3]. For older children with HGG, treatment is similar to adult patients, with attempt at maximal resection, followed by treatment with focal radiation, often with the addition of temozolomide. Infants are often treated with intensive multi-agent chemo with the goal of avoiding or delaying radiation [4]. These treatments are rarely curative, and 70-90% of patients with pediatric HGG will die within two years of diagnosis [2].

Recent molecular profiling of pediatric HGG has further highlighted important biologic differences with adult HGG. Recurrent mutations in the histone gene *H3F3A* are seen almost exclusively in pediatric HGG, and mutations in *TP53* and the histone chaperone protein *ATRX* are seen more frequently in pediatric HGG [5, 6]. These histone mutations lead to epigenetic changes resulting in transcriptional changes of developmental genes, and highlight the unique pressures that may drive tumor growth in the developing brain [1]. In fact, molecular characterization of pediatric HGGs has documented key differences *among* different sub-populations of pediatric patients, as separated by age and location [1]. As well, treatment responses may be different, with infants possibly representing a more chemotherapy-responsive sub-group [1]. These distinctions highlight the importance of future treatments in HGG being tailored to the molecular attributes of the individual tumor of the patient.

Recent work has also documented the mutation, amplification and up-regulation of *PDGFRA* in a significant subset (15-39%) of pediatric patients with HGG [2, 3]. *PDGFRA* is amplified less frequently in adult HGG, but has been found to carry a worse prognosis in adult anaplastic astrocytoma (WHO grade III glioma) [7]. An analysis of adult and pediatric HGGs showed that *PDGFRA* amplification by FISH carried a worse prognosis in adult *IDH1*-mutated HGG, but not in pediatric HGG [8]. Previous large scale genomic studies of pediatric HGG [2, 3] have not focused on the unique prognostic and treatment implication of *PDGFRA* alterations. In order to fully characterize the impact of *PDGFRA* alterations in pediatric HGG patients, we integrated genomic data from multiple datasets and sequencing platforms to create a large pediatric HGG genomic dataset (n=290).

In order to further explore the ability to target *PDGFRA*-amplified pediatric HGG, we generated a novel pediatric HGG primary cell culture with confirmed *PDGFRA* amplification. We performed molecular characterization of the matched tumor and the primary cell culture, and describe the successful targeting of *PDGFRA* with clinically available receptor tyrosine kinase inhibitors. Our genomic analysis and *in vitro* data provide compelling evidence for the continued optimization of dasatinib delivery for pediatric HGG patients with confirmed *PDGFRA* alteration.

## RESULTS

To assess the impact of *PDGFRA* alterations on survival in pediatric HGG patients, we retrieved multiple datasets of publicly available genome-wide data available in the European Genome Archive (EGA). We then integrated multiple sequencing platforms used for these datasets to produce full somatic sequence and copy number information on 290 pediatric high-grade glioma (HGG) samples (up to age 30), including 137 diffuse intrinsic pontine glioma (DIPG) and 153 non-brainstem HGG (22 anaplastic astrocytomas (WHO grade III), 125 glioblastomas (WHO grade IV), 1 anaplastic ganglioglioma, 1 gliomatosis cerebri, and 4 high-grade glioma, not otherwise specified). Of these samples, 26 (8.9%) carried *PDGFRA* mutations, 22 (7.5%) carried *PDGFRA* amplifications, 6 (2.0%) carried both *PDGFRA* mutation and amplification, for a total of 41 samples with *PDGFRA* alterations (14.1%) (Table 1). *PDGFRA* amplification was not associated with *TP53*, *FGFR1*, *ATRX* and *IDH1* mutations by McNemar's test (P < 0.05 and kappa < 0.07 for all comparisons)). *PDGFRA* mutation was not associated with *TP53*, *FGFR1*, and *IDH1* mutations (P < 0.001 and kappa < 0.12 for all comparisons). There appeared to be a slight association between *PDGFRA* mutation and *ATRX* mutation (P=0.11 and kappa=0.17).

**Table 1:** Characteristics of pediatric HGGs with *PDGFRA* alterations

	sample ID	sex	PDGFRA mutation	PDGFRA mutation type	PDGFRA copy number	age	diagnosis	location	OS (if known)	status (if known)
***PDGFRA* mutation and amplification**	pHGG_194	F	N468S	Missense	AMP	12.9	AA	Hemispheric	4.6	DOD
	pHGG_266	M	Y288C	Missense	AMP	22.7	AA	Hemispheric	18.0	DOD
	pHGG_126	F	N659K	Missense	AMP	7.6	DIPG	Brainstem	12.7	DOD
	pHGG_127	M	T281P	Missense	AMP	7.8	DIPG	Brainstem	4.4	DOD
	pHGG_226	M	I843fs	IF del	AMP	14.8	GBM	Hemispheric	13.0	DOD
	pHGG_138	F	A341T	Missense	AMP	8.7	GBM	Midline		
***PDGFRA* mutation alone**	pHGG_64	F	543fs	IF ins	NC	5.3	DIPG	Brainstem	8.7	DOD
	pHGG_66	F	A529fs	IF ins	NC	5.3	DIPG	Brainstem		
	pHGG_175	F	A341T	Missense	NC	11.0	DIPG	Brainstem		
	pHGG_191	F	N659K	Missense	NC	12.5	DIPG	Brainstem	9.6	DOD
	pHGG_58	M	K385I	Missense	NC	5.0	GBM	Hemispheric	6.0	DOD
	pHGG_102	M	Y288C	Missense	NC	6.5	GBM	Hemispheric	9.9	DOD
	pHGG_168	F	384fs	FS	NC	10.9	GBM	Hemispheric		
	pHGG_224	F	E311fs	FS	NC	14.4	GBM	Hemispheric	16.5	DOD
	pHGG_238	F	D583fs	IF del	NC	15.8	GBM	Hemispheric	12.9	DOD
	pHGG_243	M	R491fs	IF ins	NC	16.7	GBM	Hemispheric	9.2	DOD
	pHGG_254	F	C235Y	Missense	NC	19.0	GBM	Hemispheric	21.0	DOD
	pHGG_259	F	K385M	Missense	NC	20.0	GBM	Hemispheric		
	pHGG_262	F	V561A	Missense	NC	21.0	GBM	Hemispheric	25.0	alive
	pHGG_268	F	Y288C	Missense	NC	24.0	GBM	Hemispheric		
	pHGG_269	M	D576G	Missense	NC	25.0	GBM	Hemispheric	0.1	DOD
	pHGG_272	M	C235Y	Missense	NC	27.0	GBM	Hemispheric	15.0	DOD
	pHGG_277	M	V336fs	IF del	NC	30.0	GBM	Hemispheric	27.0	alive
	pHGG_51	M	N659K	Missense	NC	4.7	GBM	Midline	6.7	DOD
	pHGG_183	F	D842fs	NonFS indel	NC	12.0	GBM	Midline	8.0	DOD
	pHGG_248	M	Y555C	Missense	NC	17.2	GBM	Midline	11.5	DOD
***PDGFRA* amplification alone**	pHGG_12	M			AMP	1.8	DIPG	Brainstem	20.9	DOD
	pHGG_46	F			AMP	4.5	DIPG	Brainstem		
	pHGG_95	M			AMP	6.1	DIPG	Brainstem	14.4	DOD
	pHGG_99	F			AMP	6.4	DIPG	Brainstem	5.5	DOD
	pHGG_112	M			AMP	7.0	DIPG	Brainstem		
	pHGG_119	M			AMP	7.2	DIPG	Brainstem	6.0	DOD
	pHGG_125	M			AMP	7.6	DIPG	Brainstem	2.8	DOD
	pHGG_158	F			AMP	10.0	DIPG	Brainstem	10.0	DOD
	pHGG_165	M			AMP	10.6	DIPG	Brainstem	10.2	DOD
	pHGG_227	F			AMP	15.0	DIPG	Brainstem	13.4	DOD
	pHGG_236	M			AMP	15.6	DIPG	Brainstem	1.2	DOD
	pHGG_178	M			AMP	11.5	GBM	Hemispheric	5.0	alive
	pHGG_242	F			AMP	16.6	GBM	Hemispheric	12.0	DOD
	pHGG_252	M			AMP	17.8	GBM	Hemispheric	8.9	DOD
	pHGG_265	M			AMP	22.7	GBM	Hemispheric	11.9	DOD

IF del = in-frame deletion; FS = frameshift; IF ins = in-frame insertion; NonFS indel = non frameshift insertion/deletion; AMP = amplified; NC = normal copy number; AA = anaplastic astrocytoma; DIPG = diffuse intrinsic pontine glioma; GBM = glioblastoma; OS = overall survival; DOD = died of disease.

**Figure 1:**
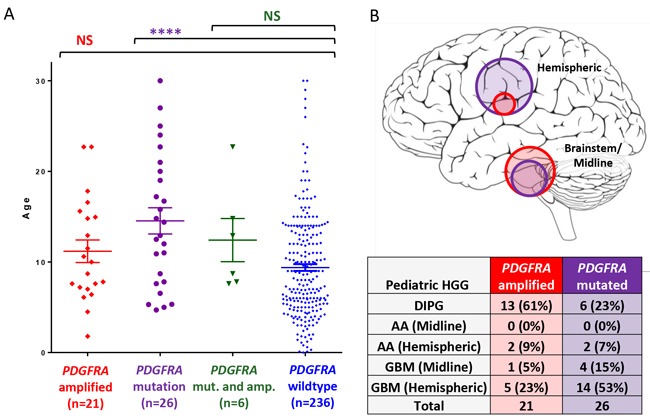
*PDGFRA* mutation is seen in older pediatric HGG patients **A.** Individual data points represent individual patients in pediatric HGG dataset (n=290), with lines representing mean and standard error of measurement (SEM). *PDGFRA* mutation (purple) was associated with older age in pediatric HGG compared to non-mutated *PDGFRA* (blue). Comparison was made using an unpaired t-test (**** = P<0.0001; *** = P<0.001, NS = P>0.05). **B.** Location of *PDGFRA* mutation (purple circles) and *PDGFRA* amplification (red circles) is represented by circle size that is proportional to frequency in pediatric HGG patients. **** = P<0.0001; *** = P<0.001; NS = P>0.05; AA = anaplastic astrocytoma; GBM = glioblastoma.

*PDGFRA* mutation, but not amplification, was associated with older age in pediatric HGG (average age 14.5 years (mutated) and 9.4 years (non-mutated); P = < 0.0001) (Figure 1A). *PDGFRA* alterations combined (mutation and/or amplification) were also seen in older patients (13.1 years) compared to *PDGFRA* wild-type pediatric HGG (9.3 years; P = 0.003). *PDGFRA* amplification was more frequently found in the brainstem; compared to *PDGFRA* mutation, which was more frequently hemispheric (Figure 1B).

**Figure 2:**
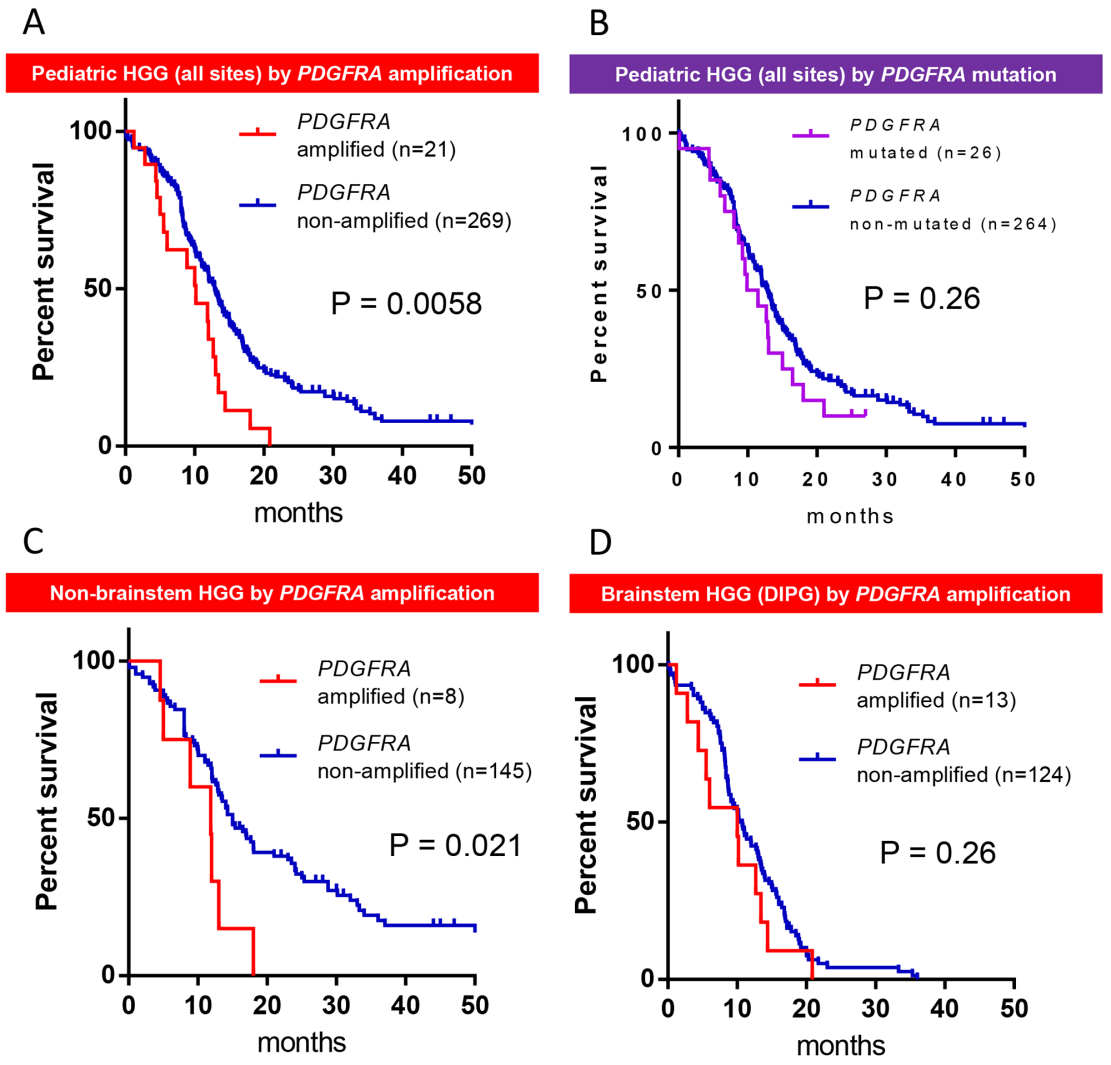
*PDGFRA* amplification is associated with worse prognosis in pediatric HGG **A.** Kaplan-Meier analysis of overall survival of 290 pediatric high-grade glioma patients from multiple integrated sequencing datasets, as divided by *PDGFRA* amplification status, with *PDGFRA*-amplified patients (red) having significantly reduced overall survival. **B.** No difference was seen in survival in pediatric HGG patients by *PDGFRA* mutational status. *PDGFRA* amplification was associated with worse prognosis in non-brainstem HGG **C.** but not brainstem HGG (DIPG) **D.**

*PDGFRA* amplification was associated with worse overall survival, when compared by Kaplan-Meier analysis (Figure 2A, *P* = 0.0058). *PDGFRA* mutation, on the other hand, was not associated with a survival difference (Figure 2B, P=0.26). When separated by anatomical location and Kaplan-Meier analysis, *PDGFRA* amplification remained associated with worse prognosis in non-brainstem HGG (Figure 2C, P=0.026), but not brainstem HGG (DIPG) (Figure 2D, P=0.26). However, when using a multivariate analysis and adjusting for age and location, *PDGFRA* mutation was correlated with worse prognosis (P = 0.026), while *PDGFRA* amplification was not (P = 0.11).

**Figure 3:**
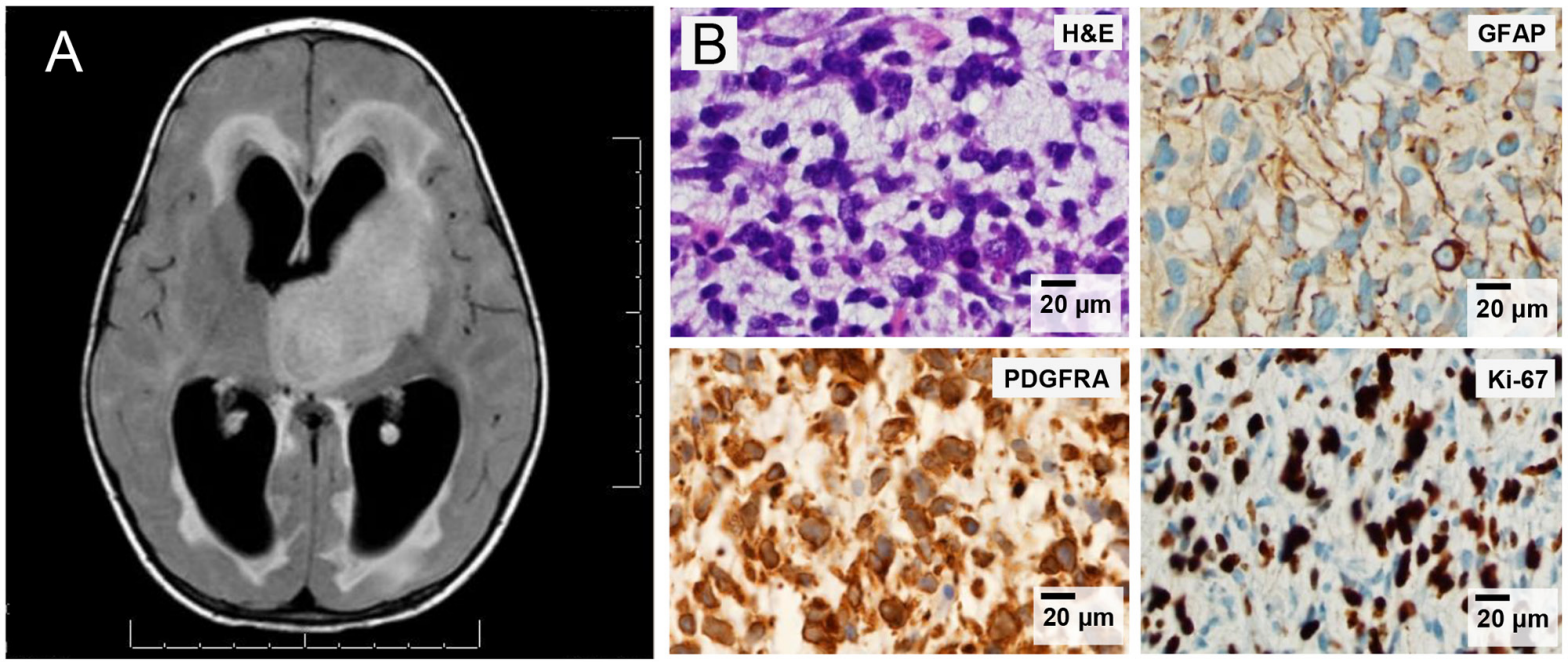
Clinical detail for infant with thalamic pediatric HGG (UMPED05) **A.** FLAIR-imaging of left thalamic tumor in two year old patient at diagnosis. **B.** Tumorhistology (Hematoxilin and Eosin) shows hypercellular glial (GFAP-positive) tumor with diffuse PDGFRA-positivity and elevated proliferation index (Ki67).

In order to further explore the biology of *PDGFRA*-amplified pediatric HGG, we performed molecular characterization and primary cell culture generation of pediatric patients presenting to the University of Michigan with HGG. A two-year old patient presented to the University of Michigan with a new infiltrative intra-cranial mass centered in the region of the left thalamus with extension into the left basal ganglia (Figure 3A). The tumor was partially resected and revealed a hypercellular spindle neoplasm, with prominent mitotic activity, without definite microvascular proliferation or necrosis. The tumor was strongly immuno-reactive for GFAP (Figure 3B), SMARCB/INI-1 was preserved, and the tumor was negative for chromogranin, synaptophysin, Cam 5.2, EMA, desmin, myogenin, neurofilament and mutant-specific IDH1 (R132H). Proliferation index (Ki-67) was markedly elevated (about 60%) (Figure 3B). These histologic findings (and the molecular profiling below) were consistent with a high-grade glioma (grade III/IV); but lack of microvascular proliferation or necrosis in examined tissue did not allow us to differentiate between WHO grade III or IV glioma.

**Figure 4:**
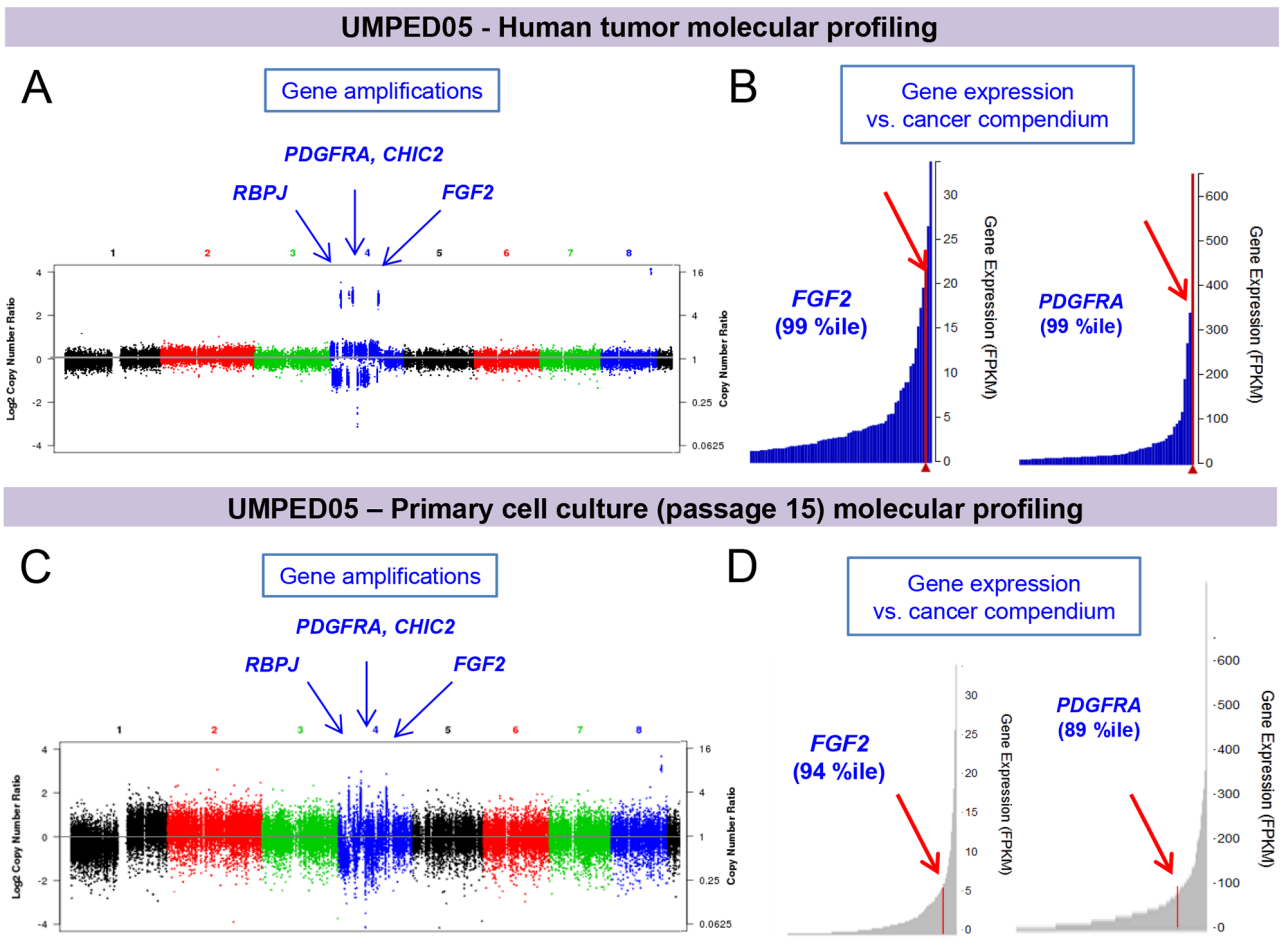
Integrative clinical sequencing results of paired tumor and primary cell culture from UMPED05 **A.** Molecular profiling shows focal gene amplifications on chromosomes 1-8, including chromosome 4 (*PDGFRA*, *CHIC2, RBPJ* and *FGF2*), and (not shown) chromosome 8 (*MYC* and *PVT1*) and 12 *(ING4*, and *ZNF384)*; and **B.** increased expression of *FGF2* and *PDGFRA*, as seen in a plot of tumor transcriptome (RNA) sequencing data. **C-D.** Molecular profiling of the cells in culture at passage 15 demonstrates retention of the key somatic events seen in the original human tumor, including amplification and outlier expression of *PDGFRA* and *FGF2*.

Integrative clinical sequencing revealed somatic tumor gene amplifications and outlier increased expression of *PDGFRA*, *MYC, PVT1*, *CHIC2, RBPJ, FGF2*, *ING4*, and *ZNF384* (Figure 4A–4B and full sequencing details in Supplementary Figure S1). The tumor showed no *H3F3A* or *TP53* mutations, which are frequently seen in this patient population [3].

In order to generate the primary tumor cell culture (UMPED05), fresh tumor tissue was collected at the time of resection, non-enzymatically dissociated, filtered and maintained in serum-containing media without any additional growth factor supplementation. The tumor cells grew adherently *in vitro.* We performed molecular profiling (exome and transcriptome sequencing) of the cells in culture at passage 15, and found them to continue to display the key somatic events seen in the original human tumor, including amplification and outlier expression of PDGFRA and FGF2 (among others) (Figure 4C–4D). The only genetic differences that we could establish in the cultured cells were sub-clonal gains of point mutations in MLL3 (~10%), which are of unclear significance (Supplementary Figure S2).

**Figure 5:**
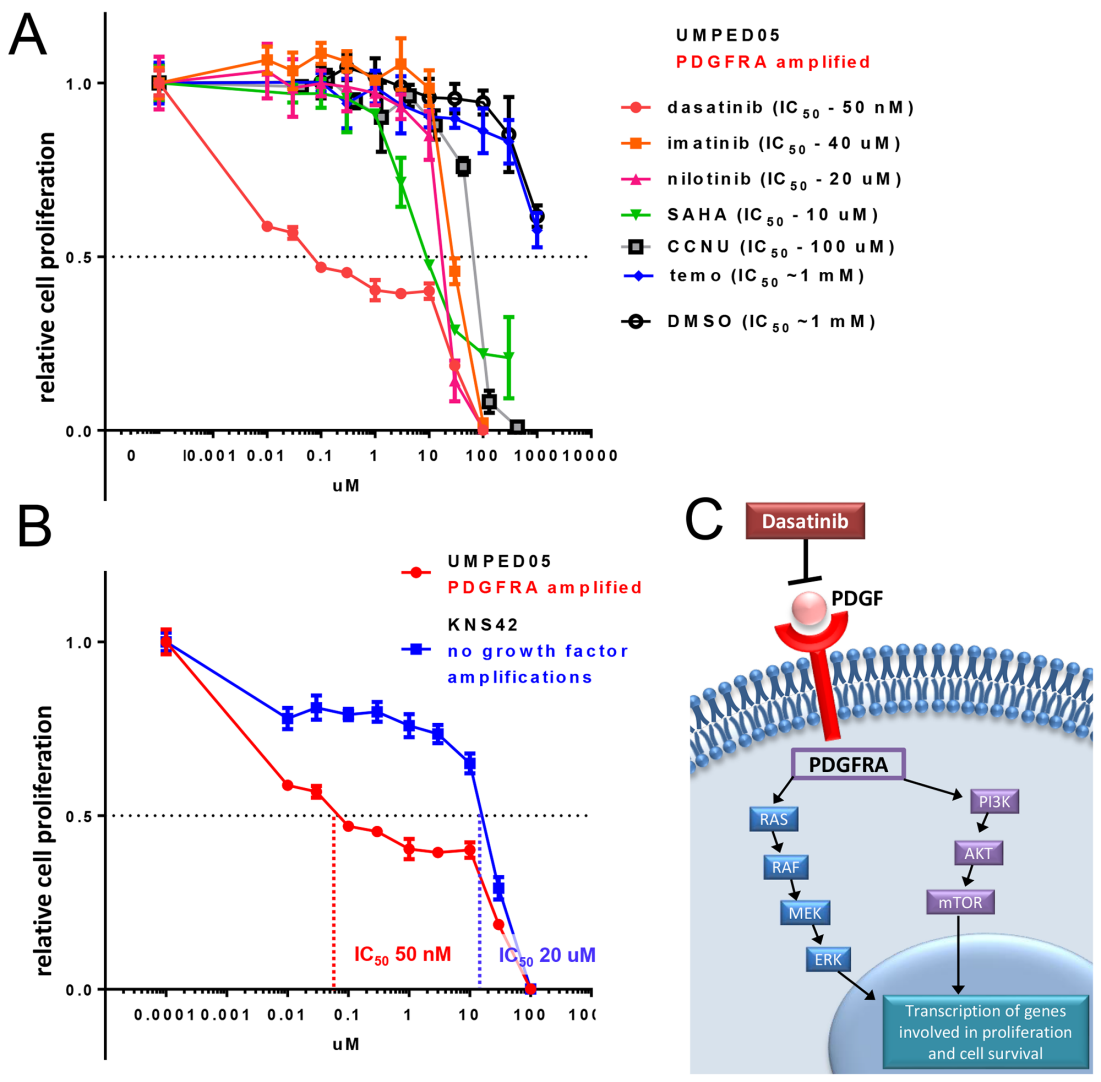
Treatment of UMPED05 with multiple chemotherapeutic agents reveals unique sensitivity to dasatinib **A.** Dose-response curves were generated by adding chemotherapeutic agents at doses ranging from 0.01-1000 μM. After 48 hours of treatment, ATP levels were measured (relative light units (RLU)) in triplicate using Cell-Titer Glo and plotted versus drug concentration. Cells were most sensitive to dasatinib treatment (in red). **B.** UMPED05 is more sensitive (lower IC_50)_ than KNS42, a pediatric GBM cell line without growth factor amplifications. **C.** Schematic representing impact of dasatinib on PDGFRA pathway.

After three passages, UMPED05 cells were treated with clinically-available tyrosine kinase inhibitors known to target PDGFR signaling, including imatinib (Novartis), nilotinib (Novartis), and dasatinib (Bristol-Myers Squibb). Of the tyrosine kinase inhibitors used, dasatinib inhibited proliferation most effectively, with the lowest IC_50_ (Figure 5A). Our cell proliferation assay correlated with cell counting assessment of viability for all agents (data not shown). We additionally treated UMPED05 cells with CCNU and temozolomide, which are used clinically in pediatric HGG patients. Temozolomide showed no significant potency (IC_50_ = ~1 mM), while CCNU showed moderate potency (IC_50_ =100 uM) (Figure 5A). Suberanilohydroxamic acid (SAHA) is a histone deacetylase (HDAC) inhibitor which has been studied in recent clinical trials of pediatric and adult high-grade glioma patients [9, 10]. Treatment of UMPED05 with SAHA showed moderate potency, with an IC_50_ of 10 μM (Figure 5A). In order to determine whether reduction in proliferation was specific to reduction in PDGFRA expression and/or FGF2 expression (UMPED05 harbors amplifications in both), we performed *in vitro* targeting of UMPED05 cells with multiple siRNAs. Reduction in proliferation was observed in transfection with one siPDGFRA, but not with two siFGF2s (Supplementary Figure S3).

Finally, we explored whether the treatment with dasatinib was specific to UMPED05. We selected another established adherent (non-growth factor supplemented) pediatric GBM cell culture (KNS42) which has been sequenced and found to have no growth factor receptor amplifications (COSMIC ID 907282) [11]. KNS42 was less sensitive to dasatinib, with a ~400 fold higher IC_50_ than UMPED05 (Figure 5B). We were unable to acquire additional HGG cell cultures with *PDGFRA* alterations, which limited our ability to confirm that the potency of dasatinib was generalizable to all glioma cells with *PDGFRA* up-regulation.

## DISCUSSION

Previous large scale genomic characterizations of pediatric HGG have shown that *PDGFRA* is a frequent tumor driver [2, 3]. However, the prognostic and treatment implications of *PDGFRA* alteration in pediatric HGG have not been fully characterized. By integrating genomic data from multiple datasets, we were able to and generate the largest pediatric HGG genomic dataset to date, with standardized somatic event calling and an adequate sample size for sub-group analysis.

In our dataset, the number of total number of pediatric patients with *PDGFRA* amplification or mutation (14.1%) was near the lower end of the range of previously published datasets [2, 3]. We report that *PDGFRA* mutation is prognostic in pediatric HGG. Previous large-scale characterizations of *PDGFRA* alterations in glioma have not reported this, either because they have focused on adults patients [7] or amplification (by FISH) alone [8]. *PDGFRA* amplification was not prognostic by multivariate analysis, which may, in part, be explained by the overlap between amplification and location (brainstem), which is highly prognostic. Non-brainstem pediatric HGG with *PDGFRA* amplification carried a worse prognosis than non-brainstem HGG without *PDGFRA* amplification, by Kaplan-Meier analysis (P = 0.021). There were no patients with *PDGFRA* amplification with reported survival beyond two years in our datasets.

Since the genomic data used in our analysis does not include treatment data, it is possible that differences in outcomes among molecular sub-groups in our analyses may have been influenced by additional treatment factors. For, example, the small number of patients (n=8) with *PDGFRA*-amplified non-brainstem HGG may have received different therapy than the larger non-amplified group. Nevertheless, prognostic difference between *PDGFRA* mutation and amplification in pediatric HGG highlights the importance of genomic characterization at the DNA level for future risk-stratification, as both may lead to changes at the RNA and protein level.

The poor prognosis seen in all sub-groups of pediatric HGG, but particularly DIPG, further highlights the clear need to improve therapies for this patient population. Our method of integrative sequencing and co-generation of a primary cell culture at the time of diagnosis of a pediatric HGG offers a feasible approach to optimize and validate personalized molecular targets.

Dasatinib is a promising agent for pediatric HGG with PDGF pathway alterations (Figure 5C). Dasatinib is an orally bioavailable tyrosine kinase inhibitor with ~60 fold greater inhibition of PDGFR signaling than earlier generation TKIs, such as imatinib [12]. While it was originally developed for targeting the BCR-ABL gene fusion, it exhibits nanomolar range activity against PDGFRA in leukemia [13]. In the previous pediatric Phase I trial using dasatinib in patients with leukemia and solid tumors, it was found to be well tolerated [14].

Dasatinib displays moderately favorable characteristics for blood-brain barrier penetration and has demonstrated efficacy in adult patients with CNS metastases of CML [15], where CSF concentrations (3 nMol/L and 20 nMol/L) were near the IC_50_ we obtained for UMPED05 (50 nMol/L) and previously published tumor cell cultures (4 nMol/L) [12]. Importantly, recent work has shown that dasatinib (or other TKI) delivery to brain tumor parenchyma may be further improved by strategies to inhibit active efflux proteins (P-glycoprotein (P-gp) and breast cancer resistance protein (Bcrp1)). Recent pre-clinical strategies have included co-administration of TKIs with agents that inhibit these proteins, including mammalian target of rapamycin (mTOR) inhibitors [16], or elacridar [17].

When used as a single agent in recurrent adult GBM, dasatinib did not show efficacy [18]. This failure in adult patients may in part be related to the increased frequency of multiple tumor driving mutations in adult HGG [19]. Pediatric tumors, including HGG, are frequently driven by fewer somatic mutations than adult tumors, and may therefore be more ideal candidates for molecularly targeted agents [20]. Additionally, optimization of future precision-medicine based therapies for HGG (adult or pediatric) may benefit from combining targeted agents for multiple key altered pathways.

For this patient (UMPED05), attempt for treatment with dasatinib was not considered until she had failed all standard therapy (surgery and multi-agent chemotherapy designed for infants with brain tumors). Unfortunately, the patient passed away shortly after the tumor progressed, and so we were unable to determine whether the patient's tumor would have responded to dasatinib clinically. Future precision-medicine based clinical trials for pediatric patients with HGG could consider up-front therapy with dasatinib for tumors with *PDGFRA* alterations.

In summary, we describe the integration of multiple pediatric HGG genomic datasets which allowed us to highlight that *PDGFRA* mutation is found in older pediatric patients and that *PDGFRA* amplification is prognostic in non-brainstem HGG. As well, we highlight the characterization and targeting of a novel pediatric HGG primary cell culture with *PDGFRA* amplification. Our work highlights the importance of genomic characterization of pediatric HGG for risk stratification and targeted therapies. Hopefully, this will be a step further in providing improved outcomes for this patient population.

## MATERIALS AND METHODS

### Human pediatric high-grade glioma dataset mutation analysis

Human pediatric glioma mutation assessment was performed using publically-available matched tumor/non-tumor genome sequencing datasets, all of which were accessed through the European Genome-phenome Archive (EGA), accession numbers EGAS00001000192,[3, 21] EGAS00001000572 [22], EGAS00001000575 [23] EGAS0000100072[24], and additional pediatric high-grade glioma samples (deposited at EGAS00001001436). Pediatric and young adult patients were included (age <30 years old at time of diagnosis), and both brainstem and non-brainstem samples were included. Somatic variants were called in alignments, using GATK version 2 and annotated for consequences with the Ensembl variant effect predictor, as previously described [6]. Copy number variations were assigned using exon level log ratios of sequence coverage in tumor/normal pairs for all known genes. Log ratios were segmented using circular binary segmentation in the DNA copy package in R version 3.1.3 and contiguous copy number aberrations were assigned using thresholds for gains/losses and amplifications/deletions based upon the average genome wide median absolute deviation in each dataset. Survival analysis was performed using Kaplan-Meier analysis (GraphPad Software, Inc.) Cox proportional hazards model was used to assess effects of somatic mutation factors, age and location on the overall survival. Kappa statistics were calculated to quantify the association between somatic alterations and McNemar's test was used to test the association. Significance is determined if P < 0.05. All analyses were conducted using SAS (version 9.4, SAS Institute, Cary, NC).

### Human tumor immunohistochemistry

Immunohistochemistry was performed in formalin-fixed, paraffin embedded tissue, 5 um sections, using antibodies against GFAP (1:3200, Dako Corporation), PDGFRA (1:100, Santa Cruz) and Ki67 (Clone 30-9 pre-diluted, Ventana Medical Systems).

### Molecular profiling human tumor tissue

Consent was obtained from the family to molecularly characterize the tumor (UMPED05) through the PEDS-MIONCOSEQ Integrative Sequencing Study, as well as to create a primary cell culture for further *in vitro* characterization and testing. Paired whole exome tumor DNA, cultured tumor cell DNA (passage 15), and germline DNA; and tumor and cultured tumor transcriptome sequencing was performed. In brief, nucleic acid preparation and high-throughput sequencing were performed using standard protocols in our Clinical Laboratory Improvement Amendments (CLIA) compliant sequencing lab. Paired-end whole exome libraries from tumor and matched normal DNAs captured by SureSelect Human All Exon V4 (Agilent, Santa Clara, CA), and transcriptome libraries from either poly-adenylated tumor RNA (PolyA+ transcriptome), or from total RNA captured by human all exon probes (capture transcriptome; Agilent, Santa Clara, CA) were prepared and sequenced using the Illumina HiSeq 2500 (Illumina Inc, San Diego, CA). Aligned exome and transcriptome sequencing reads were analyzed to detect putative somatic mutations, insertions/deletions (indels), copy-number alterations, gene fusions, and gene expression as described previously [25, 26].

### Cell culture

Human tumor primary cell culture UMPED05 was generated by harvesting tumor cells at the time of tumor resection by members of the University of Michigan Neuro-Oncology Translational Laboratory. The tumor tissue was transferred on ice and immediately dissociated using non-enzymatic cell dissociation buffer (Gibco, 13151-014), filtered and maintained in adherent cell line media [DMEM/F12 with L-Glutamine (Gibco, 11320-033)], 10% Fetal Bovine Serum (Gibco, 10437-077), Antibiotic-Antimycotic (Gibco 15240-062), and Normocin (Invivogen).

The pediatric GBM cell culture KNS42 [27] was generously provided by Dr. Alan Meeker at Johns Hopkins University, and maintained in above conditions to grow adherently.

### Treatment of cells with chemotherapy and siRNA

For high-grade glioma primary cell cultures treatment assays, cells were passaged and plated in a 96-well plate at 2,000 cells/well in triplicate per condition. At 24 hours, cells were treated with chemotherapeutic agents, siRNA, or control, and assessed for viability 72 hours later using the Cell Titer Glo Assay (Promega) [28]. ON-TARGET PLUS siRNAs (GE Healthcare Dharmacon) against PDGFRA (J003162-11; J003162-12) and FGF2 (J006695-05; J006695-06) were used according to manufacturer's protocol. Chemotherapy was administered at a range of concentrations (0.01-1000 μM) based on previously published data: temozolomide (Sigma), CCNU (Sigma), Suberanilohydroxamic acid (SAHA), imatinib (Novartis), nilotinib (Novartis), and dasatinib (Bristol-Myers Squibb). Dose response curves were plotted using GraphPad Software, and IC_50_ was calculated as the dose at which there was a 50% reduction in cell proliferation form untreated cells.

## SUPPLEMENTARY MATERIAL FIGURES


