# Supplementary material for: Characterizing and targeting PDGFRA alterations in pediatric high-grade glioma
Source: Oncotarget. 2016 Aug 25;7(40):65696–706. doi: 10.18632/oncotarget.11602 (PMC5323185; doi:10.18632/oncotarget.11602)
Supplement: Supplementary file 1 [file oncotarget-07-65696-s001.pdf]

Characterizing and targeting *PDGFRA* alterations in pediatric high-grade glioma

SUPPLEMENTARY FIGURES

UMPED05 Patient Tumor Copy Number Profile

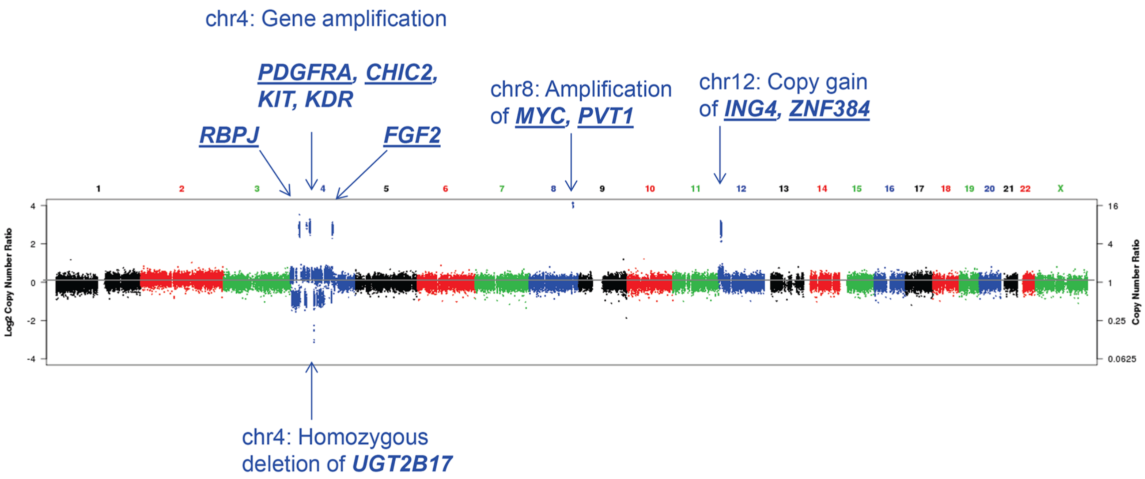

UGT2B17: UDP glucuronosyltransferase 2 family, polypeptide B17. The encoded protein is involved in the metabolism of steroids.

Supplementary Figure S1: UMPED05 Human Tumor Sequencing Results.
UMPED05 Patient Tumor Copy Number Profile

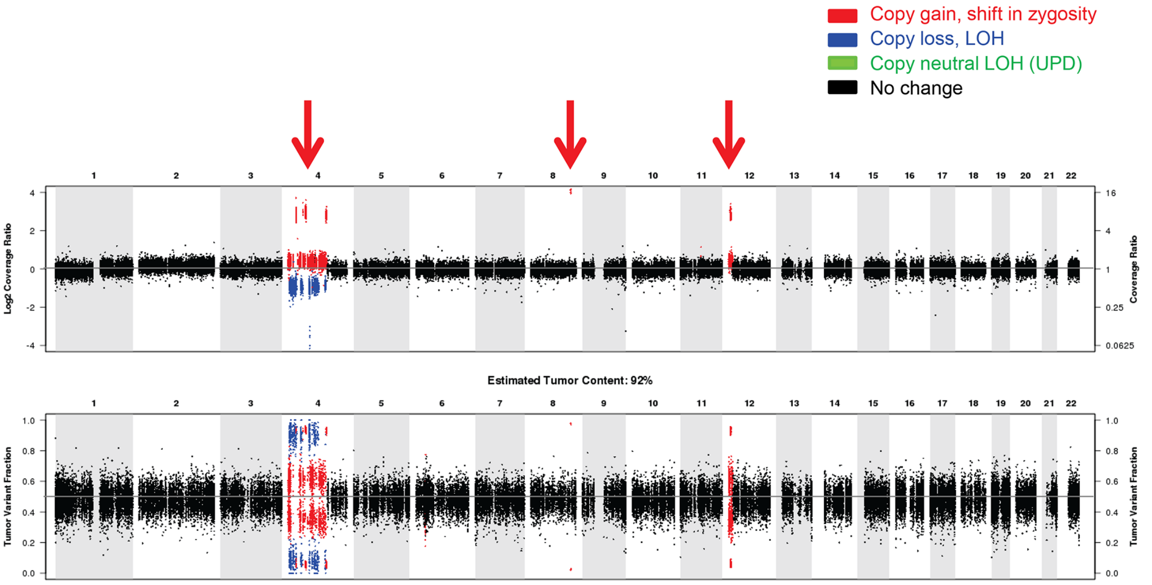

UMPED05 Patient Tumor Copy Number Profile and Loss of Heterozygosity (LOH) Plot

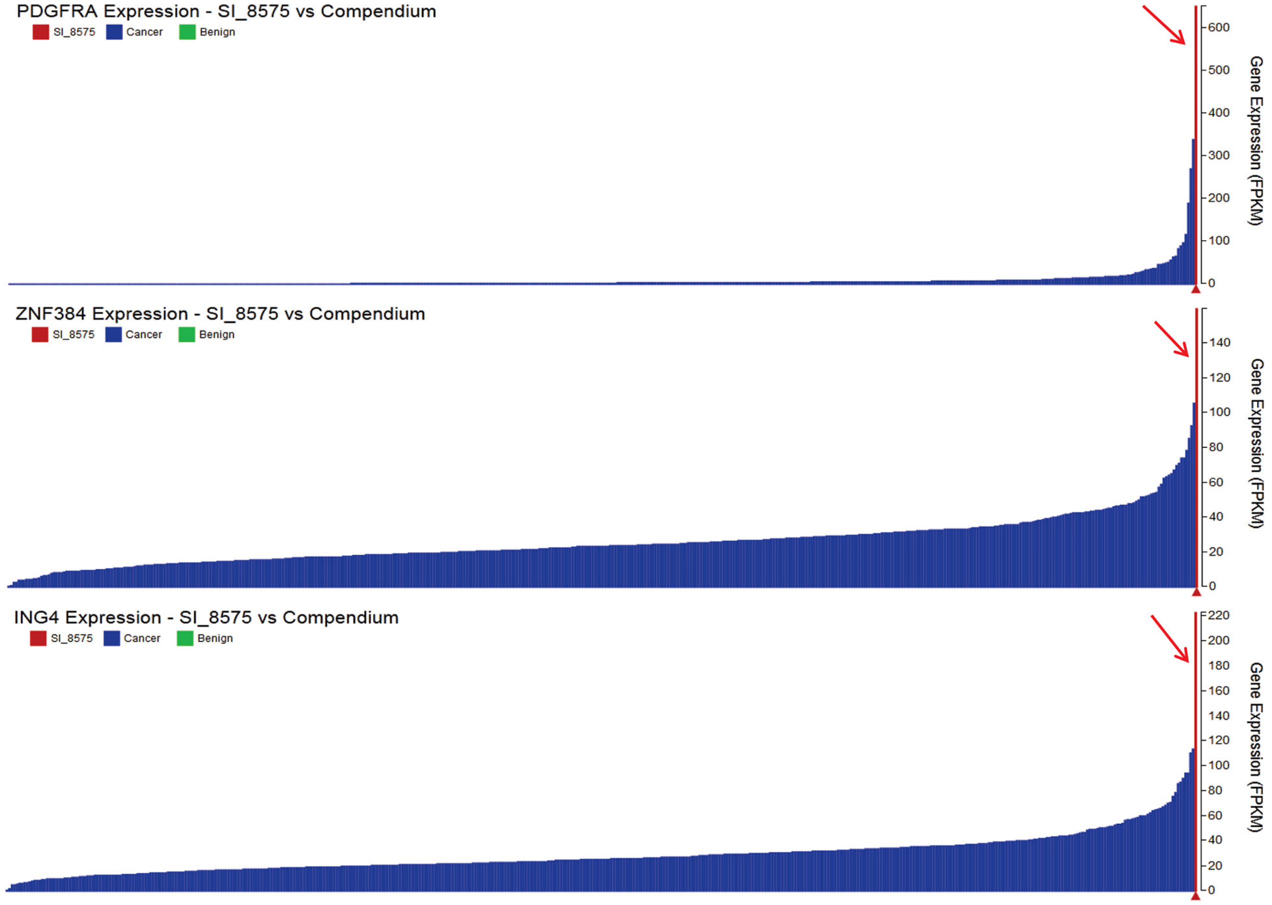

UMPED05 Patient Tumor Copy Gain Associated Outlier Expression – Part 1

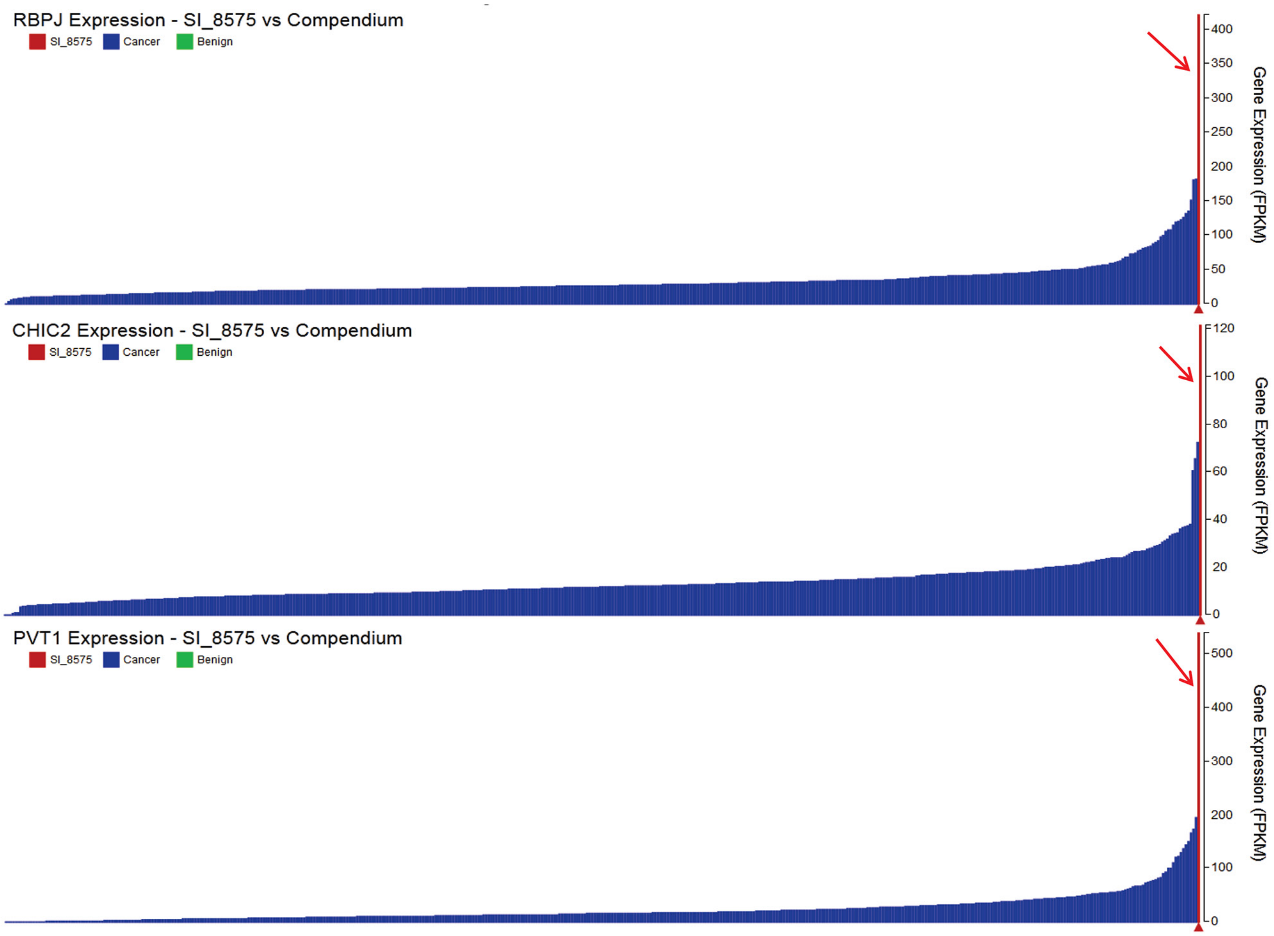

UMPED05 Patient Tumor Copy Gain Associated Outlier Expression – Part 2

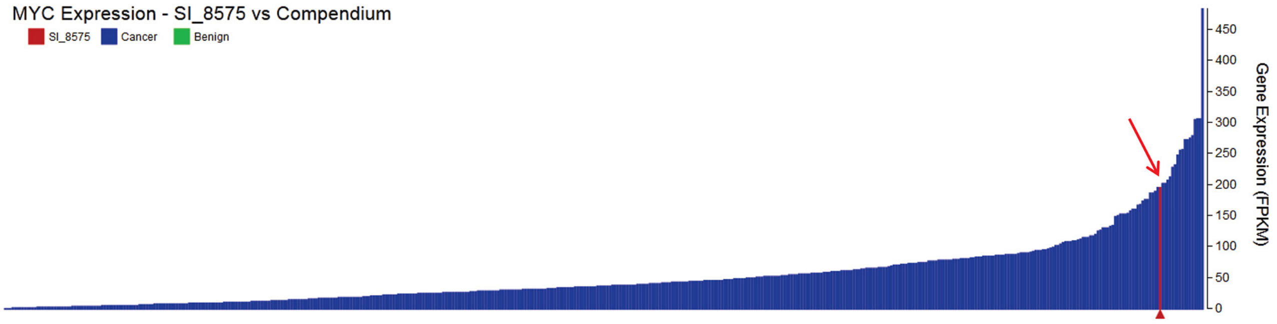

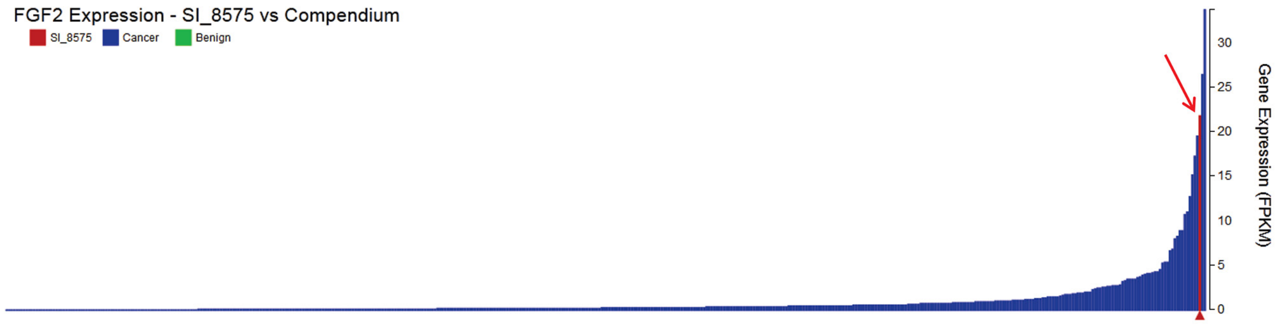

UMPED05 Patient Tumor Copy Gain Associated Outlier Expression – Part 3

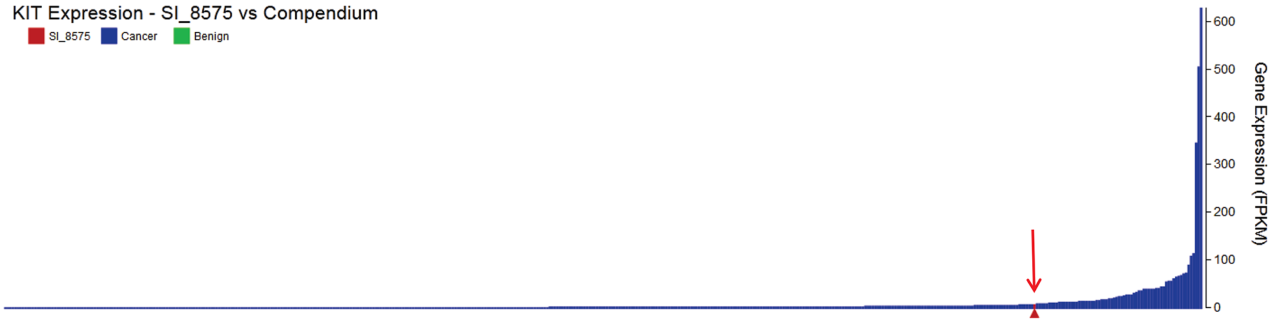

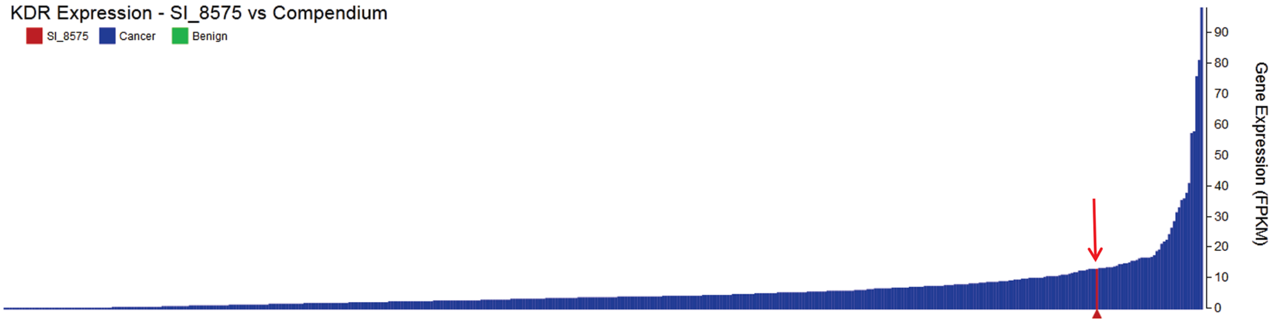

UMPED05 Patient Tumor Copy Gain Without Overexpression

Gene	Gene Set	Amino Acid Change	Chr	Coord	Ref	Var	Tumor Read Depth	Ref Reads > Q20 Tumor	Var Reads > Q20 Tumor	Var Allele Freq %	Normal Read Depth	Ref Reads > Q20 Normal	Gene Expr (FPKM)	Gene Expr percentile Compendium	COSMIC @ Pos	COSMIC +/- 6
CEP170		p.T684K	1	243329211	G	T	66	55	11	16.7	26	25	22.7	88.2	0	0
FMNL3		p.A888G	12	50041989	G	C	68	51	17	25.0	56	56	14.8	82.9	0	0
DOCK6		p.R1271C	19	11327673	G	A	252	173	76	30.5	175	174	10.1	67.0	0	0
C12orf35		p.H967Y	12	32136788	C	T	269	240	28	10.5	209	209	6.4	25.4	0	0
PER2		p.V883M	2	239162017	C	T	305	159	143	47.4	166	165	4.5	67.7	0	0
CXCL10		p.R29C	4	76943947	G	A	50	10	40	80.0	62	62	4.1	56.5	0	0
FAM18B2		p.A154S	17	15449101	G	A	102	93	9	8.8	67	64	1.8	3.2	2	2
PLIN4		p.G852C	19	4511376	C	A	85	74	11	12.9	51	50	0.1	9.7	0	0
SV2B		p.G22S	15	91769557	G	A	376	331	42	11.3	251	244	0.0	5.5	1	1

UMPED05 Tumor Somatic Mutations

Gene	Exonic Func	Chr	Start	End	Ref	Alt	Var_ Normal	Normal_ Read	Var_ Tumor	Tumor_ Read	N_var_ freq	T_var_ freq	status	AA Change
CDK11A, CDK11B	Non-frameshift insertion	1	1647893	1647893	-	TTTCTT	0	74	14	134	0	0.1	Somatic	p.R127del, insKER
PDGFRA	Non-frameshift insertion	4	55131150	55131150	-	GTG	0	128	1110	1342	0	0.83	Somatic	p.I231del, insIV

UMPED05 Tumor Insertion/Deletions

5' Gene	5' Chr	5' Coord	5 ' FPKM	3' Gene	3' Chr	3' Coord	3 ' FPKM	Gene Set	Spanning Reads	Spanning Mate Pairs	Spanning Mate Pairs w/ Fusion
SPATA5	4	124177334	188.5	LTBR	12	6490069	34.3		25	347	9
SPATA5	4	124177334	188.5	LTBR	12	6493781	34.3		73	350	36
SPATA5	4	124177334	188.5	LTBR	12	6495568	34.3		274	347	207
SPATA5	4	124177334	188.5	LTBR	12	6498003	34.3		34	347	13
WNK1	12	966414	310.9	ERC1	12	1579921	58.0		44	40	30
WNK1	12	966414	310.9	ERC1	12	1599258	58.0		226	124	144
**LRP6**	12	12300299	124.2	ETV6	12	11979335	130.3	Y	20	32	9
**LRP6**	12	12300299	124.2	ETV6	12	11979340	130.3	Y	4	32	0
**LRP6**	12	12300299	124.2	ETV6	12	11992073	130.3	Y	795	281	419
**LRP6**	12	12300299	124.2	ETV6	12	12006360	130.3	Y	2	1	1
**LRP6**	12	12300299	124.2	ETV6	12	12022357	130.3	Y	2	1	1
ETV6	12	11905512	130.3	DUSP16	12	12630948	34.7	Y	9	18	3
ETV6	12	11905512	130.3	DUSP16	12	12633279	34.7	Y	51	18	30
ANXA5	4	122605831	59.2	NECAP1	12	7933640	41.7		51	84	34
ANXA5	4	122607442	59.2	CLEC4C	12	7900073	10.6		2	5	1
CAMK2D	4	114582868	41.2	PRDM5	4	121720899	330.2		39	6	0
GRID2	4	93511436	2.2	FOXM1	12	2983690	57.7	Y	4	1	0
FRYL	4	48774631	15.7	LPHN3	4	62679606	113.5	Y	2	7	0
FRYL	4	48774870	15.7	LPHN3	4	62679606	113.5	Y	17	7	3

Chromosomes 4 and 12 fusions are amplicon associated.

LRP6 fusion lacks the signalling domain.

UMPED05 Human Tumor Fusion Candidates

Gene	Amino Acid Change	Chr	Coord	Ref	Var	Tumor Read Depth	Ref Reads > Q20 Tumor	Var Reads > Q20 Tumor	Var Allele Freq %	Normal Read Depth	Ref Reads > Q20 Normal	Var Reads > Q20 Normal	Normal Allele Freq %	1000 Genome	LOH
KIF1B	p.N1112S	1	10364578	A	G	234	124	107	46.3	170	87	82	48.5	0.0005	No
FANCL	p.L38F	2	58459232	G	A	54	28	25	47.2	27	13	14	51.9	0.0037	No
TLR2	p.P631H	4	154625951	C	A	231	120	111	48.1	156	77	79	50.6	0.01	No
PHOX2B	p.G278S	4	41747937	C	T	23	1	21	95.5	31	17	14	45.2	0.0005	Yes
APC	p.G2370V	5	112178400	G	T	324	157	164	51.1	223	106	115	52.0		No
NSD1	p.M2250I	5	176721119	G	A	317	175	140	44.4	205	116	88	43.1	0.04	No
NSD1	p.M2261T	5	176721151	T	C	322	165	151	47.8	218	118	97	45.1	0.04	No
NSD1	p.A2546T	5	176722005	G	A	273	135	131	49.3	191	90	95	51.4	0.02	No
FANCE	p.Q95R	6	35423559	A	G	109	58	51	46.8	77	38	38	50.0		No
BRAF	p.G390S	7	140487357	C	T	99	52	47	47.5	70	31	37	54.4		No
BRAF	p.E26D	7	140624426	C	A	92	34	53	60.9	83	33	48	59.3		No
MSR1	p.P346L	8	15978112	G	A	32	17	15	46.9	28	15	13	46.4	0.01	No
XPA	p.V166A	9	100449436	A	G	38	12	26	68.4	23	13	10	43.5	0.0018	No
PTPRJ	p.A293T	11	48146522	G	A	164	69	93	57.4	87	46	39	45.9	0.01	No
PTPRJ	p.I1235T	11	48185155	T	C	237	132	105	44.3	145	66	79	54.5	0.01	No
ATM	p.C107Y	11	108100039	G	A	48	28	20	41.7	39	19	20	51.3	0.0005	No
ATM	p.A112T	11	108106399	G	A	127	65	62	48.8	94	42	50	54.4	0.0005	No
ATM	p.T2438I	11	108200946	C	T	58	32	26	44.8	48	22	25	53.2	0.0005	No
SPRED1	p.V309A	15	38643456	T	C	299	157	139	47.0	193	94	94	50.0	0.0018	No
BLM	p.E140G	15	91292917	A	G	225	119	103	46.4	184	90	93	50.8	0.01	No
PALB2	p.P210L	16	23647238	G	A	241	114	126	52.5	164	97	63	39.4	0.02	No
FANCA	p.D252G	16	89869704	T	C	84	48	36	42.9	58	40	18	31.0	0.01	No
FANCA	p.A181V	16	89874756	G	A	43	20	21	51.2	32	20	11	35.5	0.03	No
FANCA	p.N8K	16	89883000	G	C	141	81	55	40.4	106	50	52	51.0	0.03	No
LAMA3	p.G3146R	18	21529813	G	A	132	67	65	49.2	84	38	45	54.2	0.0014	No
LAMA3	p.A3217T	18	21531654	G	A	183	89	90	50.3	113	63	48	43.2	0.0027	No
ELANE	p.V219I	19	856015	G	A	470	239	229	48.9	346	169	173	50.6	0.01	No

UMPED05 Human Germline Variations

Event/Gene	Aberration
Human Pathogens	Not detected
PDGFRA	Amplification and outlier expression
MYC, PVT1	Amplification and outlier expression
CHIC2	Amplification and outlier expression
RBPJ	Amplification and outlier expression
FGF2	Amplification and outlier expression
ING4	Amplification and outlier expression
ZNF384	Amplification and outlier expression
Point mutations	Detected, unknown significance
LRP6-ETV6	Gene Fusion

UMPED05 Human Tumor Sequencing Summary

Event/Gene	Alteration	Comments
Human pathogens	Not detected	
Germline variants	No pathogenic variant detected	
FGF2	Amplification and high expression	Outlier expressed in original tumor
PDGFRA	Amplification and high expression	Outlier expressed in original tumor
MYC	Amplification and high expression	Outlier expressed in original tumor
PVT1	Amplification and high expression	Outlier expressed in original tumor
RBPJ	Amplification and high expression	Outlier expressed in original tumor
MLL3	p.R248Q point mutation (13.1%)	Not detected in original tumor
MLL3	p.C302* point mutation (8.2%)	Not detected in original tumor

- The overall gene copy number landscape of cultured cells is similar to that in the original tumor.
- MLL3 mutations are subclonal based on variant frequency. Both MLL3 mutations are not detected in the original tumor.

Supplementary Figure S2: UMPED05 Cultured Cells Sequencing Summary.

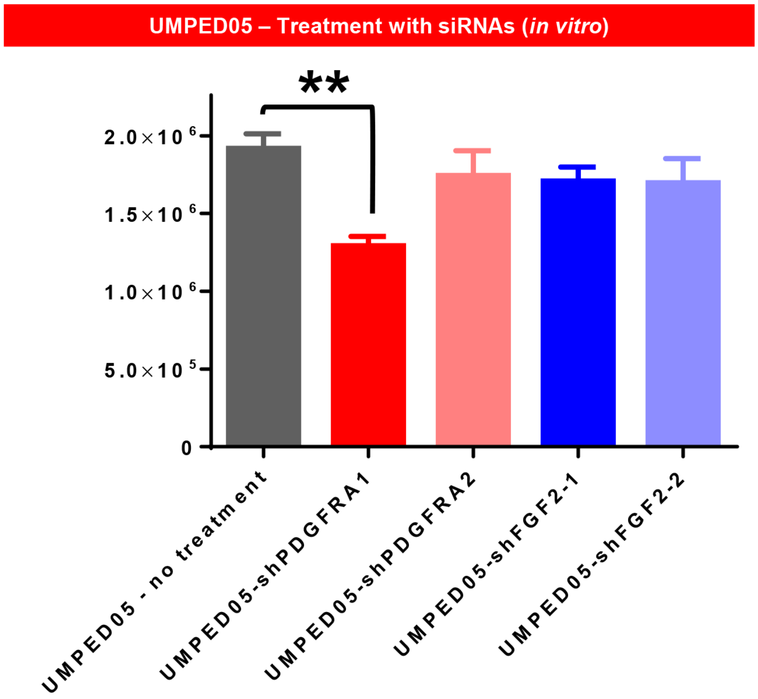

Supplementary Figure S3: Treatment of UMPED05 with siRNAs.
